# Mapping and size estimates of female sex workers in Cameroon: Toward informed policy for design and implementation in the national HIV program

**DOI:** 10.1371/journal.pone.0212315

**Published:** 2019-02-26

**Authors:** Serge C. Billong, Georges Nguefack-Tsague, Joseph Fokam, Faran Emmanuel, Shajy Isac, Raoul A. T. Fodjo, Marie Nicole Ngoufack, Sylvie Kwedi, Laure Vartan Moukam, Thomas Tchetmi, Vincent K. Tapka, Alexis Ndjolo, Zara Shubber, Nejma Cheikh, James Blanchard, Jean-Bosco N. Elat, Elizabeth N. Mziray

**Affiliations:** 1 Central Technical Group, National AIDS Control Committee, Yaoundé, Cameroon; 2 Faculty of Medicine and Biomedical Sciences, University of Yaoundé I, Yaoundé, Cameroon; 3 National HIV Drug Resistance Working Group, Ministry of Public Health, Yaoundé, Cameroon; 4 National Key Population Working Group, Ministry of Public Health, Yaoundé, Cameroon; 5 Chantal BIYA International Reference Centre for research on HIV/AIDS prevention and management, Yaoundé, Cameroon; 6 University of Manitoba, Winnipeg, Canada; 7 Faculty of Sciences, University of Yaoundé I, Yaoundé, Cameroon; 8 Association Camerounaise pour le Marketing Social, Yaoundé, Cameroon; 9 United Nations Joint Programme on HIV/AIDS, Country office, Yaoundé, Cameroon; 10 Health, Nutrition and Population, World Bank Group, Washington, United States of America; University of New Mexico Health Sciences Center, UNITED STATES

## Abstract

**Background:**

Due to high HIV prevalence among Female Sex Workers (FSWs) in Cameroon (36.5%), this population is especially vulnerable to HIV acquisition and transmission nationwide. Though being prioritized in the national HIV response, it would be relevant to generate statistics on the number of FSWs in order to guide HIV interventions among FSWs. Our objective was to estimate the size of FSWs within hotspots of Cameroon.

**Methods:**

A cross-sectional study was conducted from September-November 2015 in selected cities in Cameroon: Bafoussam, Bamenda, Bertoua, Buea, Douala, Kribi, Limbé, and Yaoundé. A programmatic mapping was used, consisting of interviews with secondary key informants (KI) to identify hotspots of FSWs and their respective estimated numbers. Validation of size estimates was done by interviews with FSW at each hotspot. Size estimations in the councils mapped were extended to others not mapped using a Poisson regression model.

**Results:**

A total of 2,194 hotspots were identified: Douala (760), Yaoundé (622), Bamenda (263), Bafoussam (194), Kribi (154), Bertoua (140), Limbé (35), and Buea (26). The estimated total number (range) of FSWs was 21,124 (16,079–26,170), distributed per city as follows: Douala 7,557 (5,550–9,364), Yaoundé 6,596 (4,712–8,480), Bafoussam 2,458 (1,994–2,923), Bamenda 1,975 (1,605–2,345), Kribi 1,121 (832–1,408), Bertoua 1,044 (891–1,198), Buea 225 (185–266), and Limbé 148 (110–148). The variability of estimates among cities was also observed within the councils of each city. The national predicted estimate of FSW population was 112,580 (103,436–121,723), covering all councils of Cameroon. An estimate of 1.91% (112,580/5,881,526; 0.47%-3.36%) adult female population in Cameroon could be sex workers.

**Conclusion:**

There are considerable numbers of FSW in major cities in Cameroon. There is a need to prioritize interventions for HIV prevention toward this population in order to limit the burden of HIV sexual transmission nationwide.

## Introduction

HIV prevalence is considerably higher among sex workers compared to the general population, with an estimated 15% of HIV positive cases in the adult female population attributable to female sex work and about 106,000 attributed deaths globally [1]. Sub Saharan Africa (SSA) has the highest attributable fraction (17.8%) of HIV transmission due to female sex work, with 98,000 estimated AIDS related deaths [2]. An analysis of 16 SSA countries showed a pooled prevalence of 36.9% among sex workers; and 14-fold higher compared to other women globally [3]. In Cameroon, HIV prevalence is 3.4% [4]; with 53,057 new infections according to the National AIDS Control Committee [5]. In line with the sustainable development goals (SDGs), Goal 3, Target 3.3: *“By 2030*, *end the epidemics of AIDS*, *tuberculosis*, *malaria and neglected tropical diseases and combat hepatitis*, *water-borne diseases and other communicable diseases”*, the national response has been strengthened in 2016 and 2017, with the support of partners (Grant Project No 993 CMR-H-MoH) [6]. With a 36.5% HIV prevalence rate among female sex workers (FSW), this population plays a very important role in the overall HIV transmission dynamics in Cameroon [7], and it is a priority population for programmatic interventions [8]. Cameroon is therefore committed to improving its HIV/AIDS response towards Key Populations (KPs), including FSWs. In the National HIV/AIDS Strategic Plan, the Government of Cameroon has henceforth prioritized programmatic interventions towards HIV prevention among FSWs [5]. It has thus become essential to quantify the size of FSWs for effective planning, monitoring and evaluation of programmatic impacts nationwide. Such information is critical for decision-making and for effective HIV prevention for FSWs both at the macro and micro levels. Although there have been attempts to estimate the size of FSWs in Cameroon, the findings were on one hand limited to selected areas in cities at the macro level [9]. Although helpful for gathering particular information, these previous approaches are unable to provide reliable size estimates. However, methods that estimate population sizes (census and enumeration, nomination techniques, multiplier methods, capture recapture) do not adequately describe the distribution and locations of populations in a geographical context [10].

On the other hand, efficient programming requires information on the estimated population size for FSWs at the aggregate level as well as granular information at the local level. Therefore, a size estimate of FSWs in Cameroon would help in ensuring that prevention services are planned and provided based on local needs.

Jing *et al*., compared the multiplier method and the generalized network scale-up method; and recommends the latter when sampling frames for the general population provided accurate demographic information is available [11]. Similarly, Yu *et al*., reviewed the methodological approaches from implementing population size estimates in 13 Asian countries [12]. After showing that all sampling methods rely on theoretical assumptions that are difficult to meet in practice, a formative assessment was recommended to help inform the appropriateness and feasibility of different size estimation methods [13]. Respondent-Driven Sampling (RDS) demonstrates sensible results when used to estimate the size of known networked populations from the National Longitudinal Study of Adolescent Health, and also when used to estimate the size of a hard-to-reach population at high risk for HIV [14]. On the other hand, Johnson *et al*., provided several country examples of how service multiplier methods have been used in respondent-driven sampling surveys and provides guidance on how to maximize the use of this method [15]. UNAIDS and WHO have also provided guideline for estimating the size of populations most at risk to HIV [16]. In Cameroon, a mixture of methods including ‘wisdom of the masses’, unique object method, and service multiplier, were used for the estimation [9]. The estimation approach used in the latter study was based on programmatic experience that populations at increased risk for HIV acquisition and transmission generally congregate and/or meet clients in specific geographic settings [17]. A main difference between programmatic mapping and other KP mapping is that programmatic mapping is based on micro level, and identifies sites where programs can reach members of a KP (in this context sex workers). Moreover, size estimates of FSWs can be readily translated into outreach targets for local service delivery providers and planned interventions. The approach was originally designed by researchers at Center for Global Public Health (CGPH) of the University of Manitoba; and has been adapted for use in various countries in South and South-east Asia and in Africa. With minor changes made to adapt the approach to local contexts and research needs, the methodology could provide more valuable information on similar populations worldwide. The original approach for programmatic mapping was performed as previously reported [17], while applications of programmatic mapping and its implications were further described [18–20]. Programmatic mapping was borrowed from the Priorities for Local Aids Control Efforts (PLACE) method, first conceived in 1999 and implemented in a township in Cape Town, South Africa [21]. Its validity and reliability has been evaluated and the method promoted alongside with programmatic mapping by the Global Fund, WHO, and UNAIDS. Many studies have been carried out on programmatic mapping. Sabin *et al*., reviewed and showed that programmatic mapping was used in 71 countries for KP (FSW, MSM, PWID, transgender) and in 27 of the 71 countries, this approach was used on FSWs [22].

With the goal to set up an effective planning and implementation based on national estimates using reliable and feasible predictions in Cameroon, our study aimed at estimating the size of FSWs in order to guide the national HIV program design and implementation. Specifically we sought to: (a) estimate the total number of FSWs (population size) in selected sites; (b) develop national predictive estimates of FSWs; and (c) estimate the proportions of FSWs among adult female in Cameroon.

## Materials and methods

### Study design

A cross-sectional study was conducted from September 5^th^ to November 30^th^ 2015 in locations of Cameroon where high-risk activities related to HIV transmission occur (hotspots) among FSWs. A FSW was defined as any female who exchanges sexual activity with a man in return for money or benefits, irrespective of site of operation (street, bars, hotel, etc.).

### Study sites

Hotspots were in the following selected cities: Bafoussam, Bamenda, Bertoua, Buea, Douala, Kribi, Limbé, and Yaoundé. Even if a hotspot may have been previously mapped, it was essential to validate the hotspots by re-confirming their current status as well as identify any new hotspots that have emerged since the previous mapping.

Several criteria were used to prioritize the cities/towns for the mapping, taking into account the Government priorities for intervention towards FSWs. The criteria included: (i) the size of the city (both large and small cities); (ii) the prevalence of HIV in the city; and (iii) the presence of existing programs for FSWs that could be leveraged.

### Study procedure

The target area for mapping was defined by selecting the cities to be mapped. Once up-to-date geographical maps were acquired, the entire area under study was divided into smaller data collection units, referred to as “zones”, which were demarcated on the maps and developed using a combination of existing geographical or administrative divisions (e.g. councils/subdivision) and physical landmarks. The methodology uses two sequential steps to identify hotspots, known as locations where FSWs seek sexual partners and/or engage in sexual activity: Level 1 (L1) included systematic information gathering from secondary key informants (KIs), who were persons in direct contact with FSWs. Based on the answers provided by KIs, a list of potential hotspots was developed. Level 2 (L2) involved the validation and profiling of identified hotspots through interviews and focus group discussions with FSWs.

### Implementing team

A Technical Advisory Group (TAG) was set up to oversee the execution of this study and to support the study implementation (Decision N° 1106/D/MINSANTE/CAB/SG/STBP/NACC/SP/SPE of June 15, 2015). The TAG, headed by the NACC, consisted of members from World Bank, UNAIDS, USAID, University of Manitoba’s CGPH, local technical experts, local stakeholders (donors and non-governmental partners), representative of NGO working with FSWs. The TAG was under the supervision of the Minister of Public Health.

### Key informants

**Fig 1:**
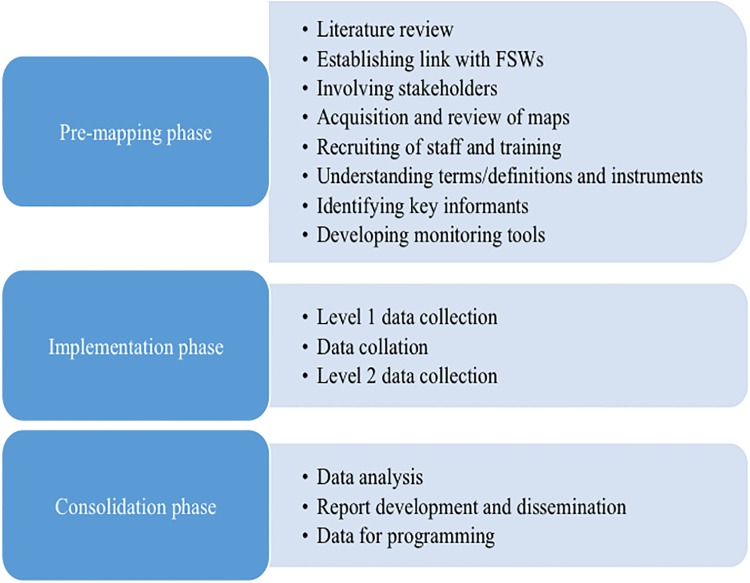
Representation of the mapping phases.

Members of the TAG agreed to the identification of relevant key informants (KIs). KIs are persons who are likely to have information on the profiles of the locations and estimates of the number of participants engaging in high-risk activity. Based on their involvement in the relevant high-risk activity mapped, KI’s were classified into two types:

Primary KIs were FSWs and were used for Level 2 data collection. Secondary Key informants were persons who are in direct contact with FSWs, or those likely to provide information on their location and are used for Level 1 data collection. Some of the following people acted as secondary KIs: MSM, Drug users (injectable and non-injectable), Taxi drivers, Local food sellers, Brothel owners/madams, Watchmen/security staff, Nanga boko (Street children), Hotel/lodge workers, Bar workers/owners/patrons, Porters, Petty shop owners, Cyber man (a controller of an internet network service shop), Drug peddlers/pushers, Pharmacists, Lottery sellers, Streets/toilets sanitary workers, Networks of MARPs; NGO staff, Health care service providers, Gov./law enforcement officials, Street families, Beggars, Public/private transport staff, Callbox men (mobile phone airtime vendor) Construction workers/laborers (Fig 1) at various places such as parks, transit stops, shopping malls, and night clubs.

### Level 1 activity

Level 1 was the first phase of field activity, in which information was collected on the various high-risk activities and individuals within each zone, by interviewing secondary KIs. This enabled the development of a comprehensive list of hotspots where FSWs congregate within each zone. The major aim was to generate a list of hotspots where such activities take place and a range of estimates (minimum and maximum) of participants in high-risk activity. For each mentioned location, KIs were asked a set of questions about the characteristics of the hotspot (public place, brothel, night club, etc.) and an estimate of the number of FSWs who can be found there (minimum and maximum). The level 1 interview was conducted at various major markets, parks, streets, etc., within a specified zone to administer questionnaires on high-risk activities taking place within that zone.

### Level 2 activity

Level 2 consists of validating information collected in Level 1, by visiting each “hotspot”, and interviewing FSWs (primary KIs) who were present at those hotspots. Hotspots were validated by determining whether they still exist or are currently inactive, and by identifying FSW typologies and estimating the number of each FSW typology at that hotspot on average and during peak times. Level 2 fieldwork also involved collecting geo-coordinates (using GPS devices) for each active hotspot validated. Using hotspot lists prepared in Level 1, teams validated hotspots by interviewing FSWs at those hotspots at the time that those hotspots were active. Following planning from the field supervisor, mobilizers identified FSW and introduced them to the team. Validation of hotspots in Level 2 was to confirm two key areas of inquiry: firstly the existence of an active hotspot and secondly a more accurate estimate per hotspot. All hotspots found in L1 were validated in L2; with interviews being conducted in all hotspots. In very few cases (8.2%), primary KIs were not available, and secondary KIs were interviewed instead. Each hotspot was visited twice, at least, before a decision to validate information from secondary KIs was made. Because KPs are often stigmatized and marginalized, the team worked with community members throughout the study by hiring these individuals as peers workers (social mobilisers), in order to ensure more access to hidden segments of sex worker and community participation.

### Data quality assurance

A monitoring and quality assurance system was designed together with a timeline to complete the data collection activities. Supervisors monitored the fieldwork by visiting each field team on a daily bases. Twenty percent (20%) of hotspots were randomly selected daily for validation by the supervisor. For both levels (L1 and L2), monitoring forms were used to assess the quality of work by site coordinator, supervisor and data entry operators. The forms were filled by the respective team members to keep track and record of the daily activities, and then were submitted to the respective line manager. About 30% of the forms were rechecked, edited for wrong entry, out of ranges values, skip patterns, irrelevant data, and spelling of text answers, then were validated by supervisors. Feedback was provided to interviewers on the basis of the errors identified in the forms. A double entry of data was performed for consistency.

### Data management and analysis

After the data set was properly coded, the entire data was entered into CSPro 6.2. Once the data entry was completed, it was exported to Microsoft Excel 2013 and IBM-SPSS Statistics (Version 21. Armonk, NY: IBM Corp.) for statistical analysis.

#### Calculation of estimates of FSWs

The crude estimates were obtained as an average of minimum and maximum number of FSWs on normal/peak days, and were adjusted to account for mobility (duplicates) of FSWs. For example, if FSWs are solicited at two or more hotspots, the number of FSWs estimated for the geographic unit that contains those hotspots will increase [23]. For a zone *i*, let *C*_*i*_ be the crude (unadjusted) estimate of FSWs, obtained by summing the estimate of FSWs from each hotspot of the zone; *P*_*i*_ the probability of a FSW operating in several hotspots, *M*_*i*_ the mean number of places where a FSW operated. The adjusted estimate *N*_*i*_ (accounting for the mobility of FSWs) was derived using the following simple mathematical function: *N*_*i*_ = *C*_*i*_(1 − *P*_*i*_)+*C*_*i*_*P*_*i*_/*M*_*i*_. Estimates from all zones in a city were subsequently summed up to generate estimates for the whole city. This size estimation formula was initially developed by the University of Manitoba, and adopted in programmatic guidelines by Global Fund in 2010; and this formula was previously used [24].

#### Predicting national estimates of FSWs

Council-wise FSW population size estimates were obtained using a Poisson regression modeling from the mapped councils. The dependent variable was estimates of FSWs (*E*_*i*_); while the square root of adult population ($A_{i}^{1/2}$) and logarithm of adult population (*Log*(*A*_*i*_)) were used as independent variables (predictors) from the 26 councils of the eight cities (the index *i* was for each council). The national estimate was obtained as the sum of predicted estimates of FSWs of each of the 360 councils in Cameroon (including the 26 councils used for estimation). The predicted interval for the national estimates (*NE*) was obtained as $NE\pm 2\sigma \sqrt{m}$ [24] where *σ* was the standard deviation of the predicted estimates of each of the *m* councils (*m* = 360); $\sigma \sqrt{m}$ was the standard deviation of a sum, and $\sigma /\sqrt{m}$ the standard deviation of a mean. Residuals from the model diagnostics showed that predictions from cities mapped were close to the estimates obtained from the mapping.

### Developing hotspot maps

The entire data, which has been collected, validated and triangulated, was displayed on zonal maps that were later merged into city maps. A combined city map was further developed using the geo-coordinates collected at Level 2 to show the spatial distribution of FSW population and its estimates. For purposes of confidentiality, hotspot maps for every city developed as part of this study were only used for programmatic perspectives. The council/sub-division maps were also developed (please contact National AIDS Control Committee (NACC) to access data, more information within the Data Availability Statement in order to identify hotspots (Fig 1).

Fig 1 shows the full representation of the programmatic methodology, from literature review to report development and dissemination.

### Ethical considerations and safety of FSWs

Ethical clearance for the study was obtained from the “*Comité National d’Ethique de la Recherche pour la Santé Humaine”* (N° 2015/07/617/CE/CNERSH/SP of July 17 2015). Administrative authorizations were obtained from each local representative of the Minister of Public Health for the cities involved. Programmatic mapping, by its nature, gathers information about places and locations where risk activities take place, so potential hazards to the field teams and respondents were addressed. Divisional Officers, Police Heads, Mayors were notified. Identity cards (temporary) signed by health or police authorities were issued to field workers to facilitate fieldwork. Considerable efforts were made to preserve the safety of respondents because sensitive questions in a public place could cause discomfort or even put respondents at risk. The teams were trained to ensure that interviews were conducted with respect to privacy. A non-identifying coding system was used to track study data while assuring non-disclosure of participants’ identity.

Recruitment of participants was conducted only after describing the study procedures and obtaining written informed consent. During the process of obtaining informed consent, participants were clearly informed that participation is voluntary and that non-participation would have no negative consequences in terms of access to programs or services. In addition, names of respondents were not asked for purposes of confidentiality and anonymity.

All survey-related materials (e.g. completed questionnaires and maps) were kept in a secure and locked cabinet at the survey office.

## Results

### Hotspots identification and distribution of secondary key informants

Table 1 shows the number of zones where FSWs can be found in various cities in Cameroon as well as the number of interviews conducted, average interview per zone and the number of hotspots identified, as compared to the Cameroon Central Bureau of the Census and Population Bureau (BUCREP) [25].

**Table 1:** Number of zones, secondary KIs (interviewers), and hotspots identified during L1 data collection.

City	Total Population*	Adult Population**	Number of zones	Number interviews (%)	Number of interviews per 1000 adult population	Average number of interviewers per zone	Number of hotspots identified (%)
Bafoussam	336,767	171,078	6	463(7.1)	2.71	77.2	228(7.9)
Bamenda	367,627	186,755	6	345(5.3)	1.85	57.5	265(9.1)
Bertoua	102,740	52,192	4	242(3.7)	4.64	60.5	157(5.4)
Buea	155,001	78,741	4	287(4.4)	3.64	71.8	63(2.2)
Douala	2,574,785	1,307,991	36	2,297 (35.0)	1.76	63.8	1,084(37.4)
Kribi	83,341	42,337	4	302(4.6)	7.13	75.5	158(5.4)
Limbé	139,527	70,880	4	275(4.2)	3.88	68.8	93(3.2)
Yaoundé	2,526,629	1,283,528	36	2,356(35.9)	1.84	65.4	854(29.4)
Total	6,286,417	3,193,500	100	6,567(100.0)	2.06	65.7	2,902(100.0)

* Cameroon Central Bureau of the Census and Population Bureau (BUCREP), 2015 estimates;

**Adult: 15–64 years; L1: level one; KI: key informants.

Cities with larger populations had the larger number of zones. Of the 100 zones, Douala and Yaoundé each had 36 zones while Bafoussam and Bamenda each had 6 zones and finally, Limbé, Bertoua and Kribi had 4 zones each.

Douala was the city with the largest number of hotspots identified (37.4%) and Yaoundé, having comparable number of interviews as Douala, had less hotspots (29.4%). Buea had the lowest number of hotspots (2.2%). The average number of interviews among the cities included in this study varied between 57.5 in Bamenda and 77.2 in Bafoussam.

**Fig 2:**
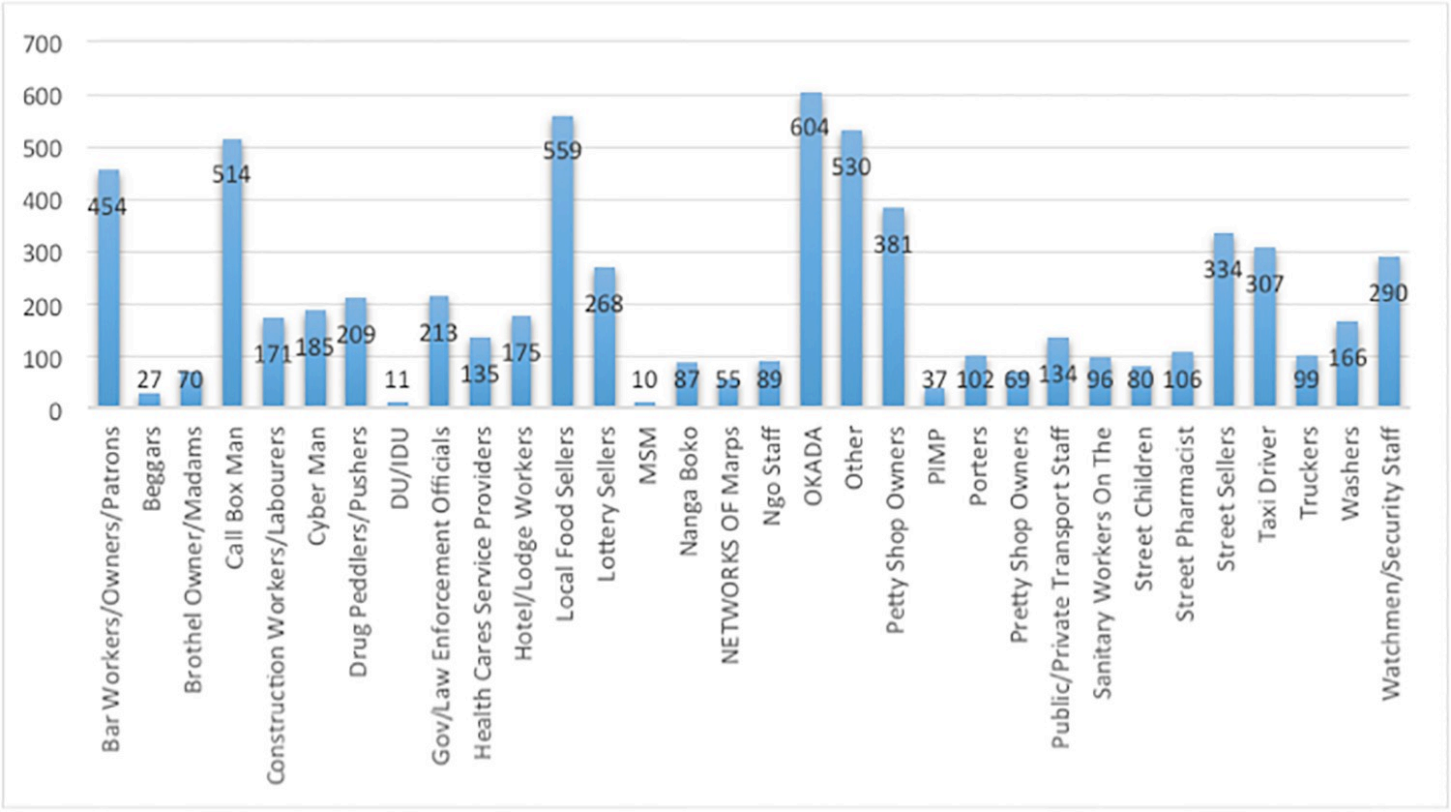
Number of L1 interviewers per type of secondary key informants. L1: level one.

Secondary KIs were mainly constituted of commercial motorcycle *(Okada)* riders (604, 9.2%), local food sellers (559, 8.5%), callbox men (514, 7.8%), bar workers/owners/patrons (454, 6.9%), petty shop owners (381, 5.8%), street vendors (334, 5.09%), and taxi drivers (307, 4.7%), as shown in Fig 2.

#### Estimation of FSWs

Of the 2,902 hotspots identified in L1; 285 (9.8%) have never been active, 597 (20.6%) have been active but no longer exit; while 2,020 (69.6%) were active; 174 new hotspots were identified; giving a total of 2,194 hotspots identified in L2. Thus, nearly 8% of hotspots were new (174/2,194).

Table 2 shows that the number of hotspots in every city was: Douala (760, 34.6%), Yaoundé (622, 28.4%), Bamenda (263, 12.0%), Bafoussam (194, 8.8%), Kribi (154, 7.0%), Bertoua (140, 6.4%), Limbe (35, 1.6%), and Buea (26, 1.2%); with disaggregated data from councils showing significant heterogeneity. Table 2 shows the number of hotspots, estimated number of FSWs on usual days and on peak days in each of the mapped cities. It is important to note that on peak days, the estimated total number (with range) of FSWs was 21,124 in all cities mapped, with Douala having the largest number of FSWs (7,557:5,750–9,364) and the lowest number registered in Limbé (148:110–148). On normal days, the lowest number of FSWs was found in Limbé (124, 91–156) while the highest number was found in Douala (3,960: 3,023–4,896). Table 2 also shows that heterogeneity in the Parameters for adjusting for mobility (duplicates) of FSWs; with the mean varying from 1.22 in Douala to 2.59 in Bamenda. Bamenda (2.59), Bertoua (2.51) and Limbé (2.31) are cities with higher mobility of FSWs.

**Table 2:** Estimated number of FSWs per city with mobility parameters.

		Parameter for adjusting for mobility (duplicates)		Estimated number of FSWs on usual days			Estimated number of FSWs on Peak days		
City	Number of hotspots (%)	Mean (*M*_*i*_)	Probability (*P*_*i*_)	Minimum	Maximum	Average	Minimum	Maximum	Average
Bafoussam	194 (8.8)	1.43	0.67	1,244	1,837	1,540	1,994	2,923	2,458
Bamenda	263 (12.0)	2.59	0.96	846	1,372	1,109	1,605	2,345	1,975
Bertoua	140(6.4)	2.51	0.90	500	691	596	891	1,198	1,044
Buea	26(1.2)	1.46	0.71	91	156	124	185	266	225
Douala	760(34.6)	1.22	0.85	3,023	4,896	3,960	5,750	9,364	7,557
Kribi	154(7.0)	1.78	0.89	499	922	711	832	1,408	1,121
Limbé	35 (1.6)	2.31	0.81	77	150	114	110	186	148
Yaoundé	622 (28.4)	1.52	0.61	2,455	4,451	3,453	4,712	8,480	6,596
Total	2,194 (100.0)	1.44	0.77	8,735	14,475	11,607	16,079	26,170	21,124

Per capita FSWs estimates; FSW: female sex workers.

The number of FSWs per 1000 adult males was particularly higher in Kribi (55.39), Bertoua (41.85), and Bafoussam (30.06), as shown in Table 3. Variations were also observed between rural and urban settings of the cities. In Urban settings, Bertoua (44.75), Bafoussam (35.74), and Bamenda (26.22) registered the highest numbers, while in the rural settings, highest numbers were registered in Bertoua (38.27), Bamenda (16.67), Bafoussam (14.82). Considering rural and urban settings of the same city, only Bafoussam registered a greater difference.

**Table 3:** Comparison of estimates between cities.

City	Type	Male Population	Female Population	Total	Adult Male Population	Adult Female Population	Adult Population	FSW Estimates	FSW Per 1000 Adult Male	FSW Per 1000 Adult Female
Bafoussam	Rural	43,098	48,325	91,423	22,200	24,243	46,443	329	14.82	13.57
	Urban	118,044	127,300	245,344	59,575	65,059	124,635	2,129	35.74	32.72
	Total	161,142	175,625	336,767	81,775	89,303	171,078	2,458	30.06	27.52
Bamenda	Rural	77,947	79,868	157,815	38,321	41,849	80,170	639	16.67	15.27
	Urban	101,986	107,826	209,812	50,947	55,637	106,584	1,336	26.22	24.01
	Total	179,933	187,694	367,627	89,269	97,486	186,755	1,975	22.12	20.26
Bertoua	Rural	23,656	22,397	46,053	11,183	12,212	23,395	428	38.27	35.05
	Urban	28,946	27,741	56,687	13,765	15,032	28,797	616	44.75	40.98
	Total	52,602	50,138	102,740	24,948	27,244	52,192	1,044	41.85	38.32
Buea	Urban	77,427	77,574	155,001	37,638	41,103	78,741	225	5.98	5.47
Douala	Urban	1,293,692	1,281,093	2,574,785	625,220	682,771	1,307,991	7,557	12.09	11.07
Kribi	Urban	41,679	41,662	83,341	20,237	22,100	42,337	1,121	55.39	50.72
Limbe	Urban	68,316	71,211	139,527	33,881	36,999	70,880	148	4.37	4.00
Yaoundé	Rural	59,580	60,179	119,759	29,080	31,757	60,838	99	3.40	3.12
	Urban	1,208,017	1,198,853	2,406,870	584,446	638,244	1,222,690	6,497	11.12	10.18
	Total	1,267,597	1,259,032	2,526,629	613,526	670,001	1,283,528	6,596	10.75	9.84
	**Total**	3,142,388	3,144,029	6,286,417	1,526,493	1,667,007	3,193,500	21,124	13.84	12.67

FSW: female sex workers.

**Fig 3:**
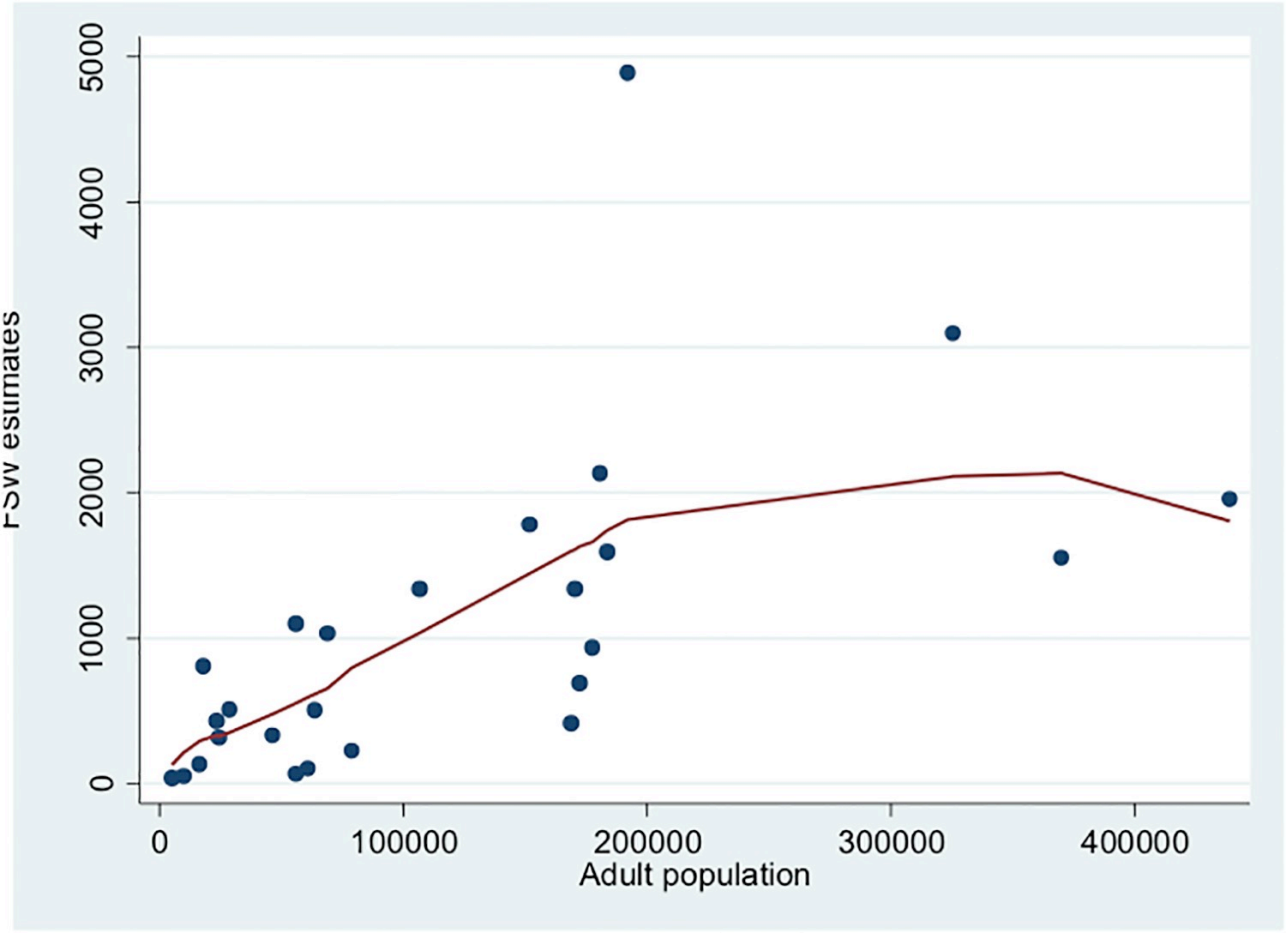
FSW estimates against adult population in councils of selected cities. FSW: female sex worker.

Fig 3 shows that FSW estimates increased with the size of adult population. The local linear smooth plot using Cleveland’s tricube weighting function [26] shows that FSW estimate is not related to adult population through a simple linear regression model. This may be due to the presence of three possible 3 outliers. We have thus fitted a Poisson regression model to 26 councils that led to the following estimates of the coefficients: Constant = -2.724; -0.002 for the coefficient of square root of adult population ($A_{i}^{1/2}$), and 0.87 for the coefficient of the logarithm of adult population (*Log*(*A*_*i*_)).

This outcome led to a national predicted estimate of FSWs of 112,580 (103,436–121,723), covering all councils of Cameroon. Predictions showed that 112,580 out of 5,881,526 adult female population aged 15–64 years were FSWs, thus approximately two percent (1.9%: 0.47–3.36) of adult female population in Cameroon could be sex workers. The estimate was 55,845 (43,997–67,694) for urban councils, indicating that half of FSWs are in urban areas.

### Prediction of FSWs estimation in the studied sites

The predicted FSWs per 1000 adult male varied from 13.91 (Littoral) to 30.88 (East). The regions of South and East had the highest number of FSWs per adult female with 27.47 and 28.28 respectively, then highest percentages of FSWs (see Table 4).

**Table 4:** Prediction of FSWs estimation per region.

Region	Number of councils	Total Population	Adult Population	Adult Female	Adult Male	Predicted FSWs size	1000 Adult Female	1000 Adult Male
Adamawa	21	1,200,970	610,093	318,468	291,625	7,059	22.17	24.21
Centre	70	4,159,492	2,113,022	1,102,997	1,010,025	18,384	16.67	18.20
East	34	835,642	424,506	221,592	202,914	6,266	28.28	30.88
Far-Nord	47	3,993,007	2,028,448	1,058,850	969,598	20,674	19.53	21.32
Littoral	34	3,354,978	1,704,329	889,660	814,669	11,331	12.74	13.91
Nord	21	2,442,578	1,240,830	647,713	593,117	11,092	17.12	18.70
Nord-West	34	1,968,578	1,000,038	522,020	478,018	11,618	22.26	24.30
West	40	1,921,590	976,168	509,560	466,608	11,639	22.84	24.94
South	29	749,552	380,772	198,763	182,009	5,460	27.47	30.00
South-West	31	1,553,320	789,087	411,903	377,184	9,056	21.99	24.01
Total	361	22,179,707	11,267,291	5,881,526	5,385,76	112,580	19.14	20.90

FSW: female sex workers

### Locations of FSWs

The hotspots appeared to be unevenly distributed across and within cities and often regrouped into clusters. Most hotspots were located in the urban area of each city. The maps generated from this study found that most cities had hotspots concentrated in the commercial centers and along the major roads and highways leading in and out of the cities. In larger cities such as Douala, this pattern was persistent but did not preclude activities from being dispersed to other areas of the city. In Douala and Yaoundé, some hotspots were also located in rural settings.

## Discussion

Estimating the size of FSWs in a country with generalized HIV epidemics is of great importance to set-up programmatic interventions toward a better prevention of HIV transmission within such a high-risk population within a resource-limited setting context. To our knowledge, this study is the first in Cameroon to estimate the size of FSWs at micro (hotspot) level for programming purposes. The comparison of the size of FSWs between different cities was better appreciated by calculating the ratio of FSWs per 1000 adult male/female. These estimations showed the highest ratio of FSWs in Kribi (55.39) and Bertoua (41.85). Kribi is a touristic city visited and inhabited by many wealthy foreigners because of popular beaches.

Many large scale developmental projects are currently being implemented in Kribi, such as its port. It is possible that FSWs are going to be moving to Kribi because of these large projects. Many hotspots are found in the area neighboring an oil producing factory showing a concentration of FSWs. Sex work does not seem to be stigmatized in Kribi, as culturally, the population may consider this work as an actual profession. On weekends, many FSWs residing in Douala and Yaoundé may go to Kribi to solicit clients.

Many large scale developmental projects (such as the Lom Pangar Dam) are being implemented in Bertoua. There are also many operating forestry enterprises exploiting wood in that city. Many truckers (also high risk populations) who transport goods from Cameroon to other Central African countries reside in Bertoua.

The high rate of FSWs per 1000 adult male (30.06) in Bafoussam may be explained considering that, in the west region, funerals and other traditional ceremonies are usually organized on weekends. Thus, many FSWs may use this opportunity to congregate in Bafoussam in search of clients.

Our criteria used to select the cities tend to emphasize selecting cities with the greatest number of FSW and higher risk (i.e. where normally FSW programs would be implemented). This can potentially lead to bias in selecting the cities with larger populations of FSW for mapping, which can overestimate the national FSW population when generalizing from the 8 cities to the rest of the country, even taking into account population proportions. This trade-off between practical criteria to city selection and generalizability has been taken into account by using the square root of adult population ($A_{i}^{1/2}$) and logarithm of adult population (*Log*(*A*_*i*_)) as covariates in a Poisson regression; in order to avoid an exponential growth. Odek *et al*., used a linear regression in Kenya, with the estimates of FSW per 1000 adult population as dependent variable; adult population and its square as two independent variables [24]. The density of FSWs (per capita) being very different across cities in Cameroon is clearly an indication that in addition to population size, many other variables should enter into play as other predictors when estimating the size of FSWs. Some of these variables include: (a) prevalence of HIV, (b) whether the area is urban or rural, (c) being a touristic site, (d) its economic opportunities, and (e) location along a major highway. Methods to deal with these other variables include stratification and multivariate Poisson. In either case, the sample size (n = 26) is insufficient to carry out such analyses.

The national predicted estimate (based on councils level) of FSWs was 112,580 (103,436–121,723); nearly two percent (1.91%: 0.47–3.36%) of adult female population in Cameroon.

Papworth *et al*., found an estimate of 98,102 (59,914–135,978) FSWs, aged 15–49 years, in the entire country; and 38,582 (23,563–53,477) in urban areas (nearly forty percent of FSW are in urban areas) [9]. The cities involved were Bamenda, Bafoussam, Bertoua, Douala, Kribi, Ngaoundere and Yaoundé. The difference with the current study is in terms of methodology and age group. It should be noted that Papworth et al used a mixture of methods including ‘wisdom of the masses’, unique object method, and service multiplier. Unlike programmatic mapping whose methodology is based on hotspots (micro level), Papworth et al. focused at macro level with 15–45 years as age group. Furthermore, Papworth *et al*., estimated that 1.88% (1.15–2.61%) of the female population in Cameroon are FSWs comparable to 1.91% (0.5–3.43%) obtained from the current study for adult female [9]. Odek *et al*., found a national urban FSW population estimate of 138,420 (107,552–169,288) covering all towns with a population of over 5,000 in Kenya [24]. They also found that approximately 5% of the urban female population of reproductive age in Kenya could be sex workers [24]. For this study, out of 26 councils, 5 were rural: Bafoussam 3, Bamenda 1, Bamenda 3, Bertoua 2, and Yaoundé 7; thus the sample of cities here is mixture of urban and rural. Since the predictions are based on estimating the model for only 26 councils for eights selected cities (not on hotspots level), fits of this model could be improved if more councils were mapped.

The South and East regions have higher predicted proportions of FSWs with 2.75% and 2.83% respectively. These results may partially explain the high HIV prevalence in these regions; 7.2% in the South region and 6.3% in the East region (National Institute of Statistics and ICF International). These results can serve in predicting the number of condoms to be distributed per region, as well as the minimum package of interventions for each region.

### Evidence-based programmatic implications from the study

The current study estimated the number of FSWs that could be reached by service delivery providers working with FSWs in the eight cities with the highest priority for HIV prevention in the country. Our findings are therefore of relevance for implementing interventions following the Continuum of Prevention, Care and Treatment of HIV/AIDS with Key Populations (CHAMP) project in Cameroon.

Within the UNAIDS’ 90-90-90 targets, Cameroon will considerately increase the number of people screened for HIV, and the ART program is expected to double the number of people receiving antiretroviral treatment. The results are expected to identify priority areas of intervention and guide the choice of community-based organizations (CBO) responsible for the implementation. Recommendations will serve for other HIV-related studies such as integrated biological and behavioral surveillance (IBBS) studies in the country. Because FSWs constitute a mobile population, planned interventions should start as early as possible, using current mapping as a routine exercise in order to help NGOs to achieve effective interventions. Mapping of other councils should continue in order to have reliable predictive estimates of the size of FSWs at the entire national level. Implications of our findings therefore include providing: (a) denominators that can be used for setting coverage targets and program reach; (b) critical information that can be used as indicators for implementing agencies; and (c) sampling frame for operational studies. The Ministry of Public Health in Cameroon has endorsed these estimates of FSWs for planning, budgeting, monitoring and evaluation of interventions in Cameroon in subsequent years [27–29].

### Study limitations

Some limitations could be reported from our study. For instance, our estimation was based on information from KIs, which is an approach less accurate compared to a census design, which is generally unfeasible in Cameroon since FSWs are stigmatized and they fear being recognized by family members and friends. Also because sex work is illegal, they also have fear of being arrested by the police, thus making it difficult to reach them. In addition, this study could not capture hidden FSWs who do not operate in public places (e.g. home-based FSWs), virtual places, and internet-based facilities. The adult population alone does not suffice in sizing FSWs in a city. Thus, predictions of this study can only be considered proxy of the true size of FSWs in Cameroon. The entire area under study was divided into smaller data collection units, referred to as “zones”, in order to generate findings closer to the census approach and to maximize the probability of not missing any hotspot. Finally, the sample size was small to carry out multiple Poisson regression with many other covariates.

The formula used herein for estimation accounts for mobility of FSW between hotspots in a zone. However, it does not make any adjustment for mobility between cities, which infers likelihood for double counting. In contrast, this bias is highly minimal because very few FSWs move from one city to another to find clients. Further investigations should be undertaken to account for such evidence and their programmatic implications.

## Conclusions

There are considerable numbers of FSWs in major cities in Cameroon. There is thus a need to prioritize interventions for HIV prevention towards these populations in order to limit the burden of HIV sexual transmission nationwide. Moreover, our findings would serve as baseline for informing the national HIV program on planning, implementation, monitoring and evaluation targeting FSWs in Cameroon.
